# Methanobactin-Mediated One-Step Synthesis of Gold Nanoparticles

**DOI:** 10.3390/ijms141121676

**Published:** 2013-11-01

**Authors:** Jia-ying Xin, Dan-dan Cheng, Lan-xuan Zhang, Kai Lin, Hong-chen Fan, Yan Wang, Chun-gu Xia

**Affiliations:** 1 Key Laboratory for Food Science & Engineering, Harbin University of Commerce, Harbin 150076, China; E-Mails: hashangdacdd@163.com (D.C.); glklkk@126.com (K.L.); Fanhongchen1@sina.com (H.F.); zhanglanxuan2008@yeah.net (L.Z.); wyan@hrbcu.edu.cn (Y.W.); 2 State Key Laboratory for Oxo Synthesis & Selective Oxidation, Lanzhou Institute of Chemical Physics, Chinese Academy of Sciences, Lanzhou 730000, China; E-Mail: cgxia@licp.ac.cn

**Keywords:** methanobactin, methanotrophs, gold nanoparticles, hydroquinone, monodisperse

## Abstract

Preparation of gold nanoparticles with a narrow size distribution has enormous importance in nanotechnology. Methanobactin (Mb) is a copper-binding small peptide that appears to function as an agent for copper sequestration and uptake in methanotrophs. Mb can also bind and catalytically reduce Au (III) to Au (0). In this study, we demonstrate a facile Mb-mediated one-step synthetic route to prepare monodispersed gold nanoparticles. Continuous reduction of Au (III) by Mb can be achieved by using hydroquinone as the reducing agent. The gold nanoparticles have been characterized by UV-visible spectroscopy. The formation and the surface plasmon resonance properties of the gold nanoparticles are highly dependent on the ratio of Au (III) to Mb in solution. X-ray photoelectron spectroscopy (XPS), fluorescence spectra and Fourier transform-infrared spectroscopy (FT-IR) spectra suggest that Mb molecules catalytically reduce Au (III) to Au (0) with the concomitant production of gold nanoparticles, and then, Mb statically adsorbed onto the surface of gold nanoparticles to form an Mb-gold nanoparticles assembly. This avoids secondary nucleation. The formed gold nanoparticles have been demonstrated to be monodispersed and uniform by transmission electron microscopy (TEM) images. Analysis of these particles shows an average size of 14.9 nm with a standard deviation of 1.1 nm. The gold nanoparticles are extremely stable and can resist aggregation, even after several months.

## 1. Introduction

Gold nanoparticles have received considerable attention during the past few decades because of their excellent functions in catalysis, biosensing, drug delivery and photonics [1,2]. Synthesis of gold nanoparticles with homogeneous sizes and shapes has enormous importance in nanotechnology, because of their size-dependent optical, magnetic, electronic and catalytic properties [3,4]. Although various physical and chemical methods have been developed for nanoparticle synthesis, the major challenge remains of obtaining monodispersed nanoparticles with a narrow size distribution [5]. A probable reason for this is that seed formation and growth occurs simultaneously, and sometimes, a substantial secondary population of smaller nanoparticles is formed in addition to the growth of the seeds. As a consequence, the nanoparticles are often observed to have polydispersity and a broad size distribution [6]. Currently, the most accepted size-controlled synthesis of nanoparticles is carried out by a two-step process, *i.e.*, nucleation and then successive growth of the seed particles. In the first step, a part of the Au (III) ions in solution is reduced to Au (0) atoms by a suitable reducing agent. The Au (0) atoms thus produced agglomerate to from small metal clusters, which act as nucleation centers. In the second step, the preformed seeds are put into a growth solution containing HAuCl_4_ and another reducing agent. Nucleation centers catalyze the reduction of the remaining Au (III) ions present in the adsorbed state, which promises to obtain particles of the desired size [6]. However, this two-step preparation process is complicated. There is still extensive interest in developing aqueous-based rapid one-step procedures for the synthesis of monodispersed gold nanoparticles.

**Figure 1:**
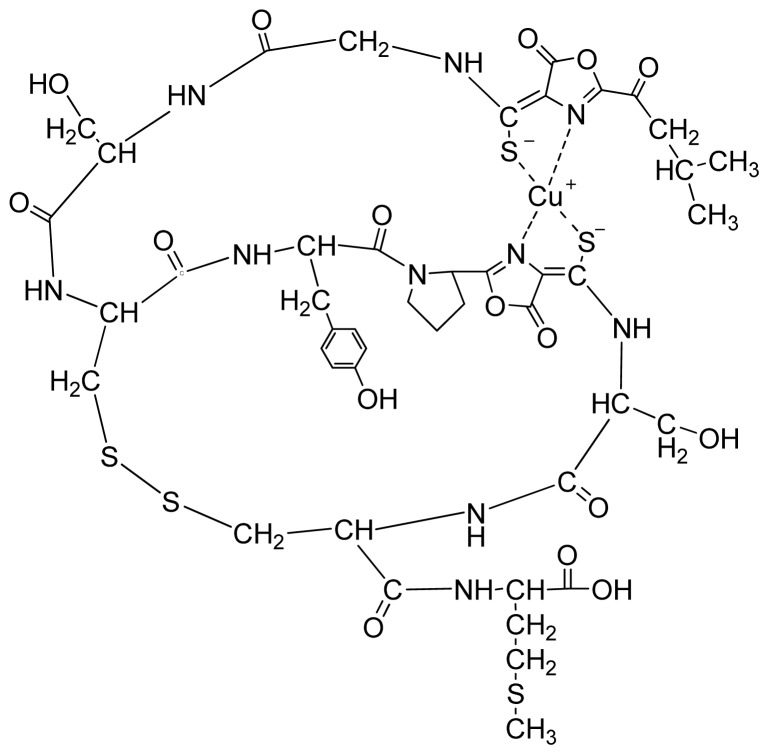
Proposed structure of Mb-Cu from *Methylosinus trichosporium* OB3b [8].

Methanotrophs are a group of ubiquitous Gram-negative bacteria that utilize methane as their primary source of energy and carbon. It is known that the amount of bioavailable copper regulates the methane monooxygenase (MMO) used by methanotrophs to oxidize methane [7]. Methanobactin (Mb) is a small, copper-binding peptide secreted by methanotrophs that extracts and uptakes copper outside of the bacterial cell. The crystal structure of copper-loaded Mb (Cu-Mb) from *Methylosinus trichosporium* OB3b revealed a 1217 Da molecule with a chemical composition of C_45_N_12_O_14_H_62_S_5_Cu (Figure 1) [8]. Mb can also bind to a number of other metals, including gold, iron, nickel, zinc, cobalt, cadmium, mercury and uranium [9]. It has been found that Au (III) can be reduced to Au (0), and then, Au (0) remains associated with the Mb. Examination of Au-Mb complexes by transmission electron microscopy (TEM) showed little to no detection of nanoparticles. However, if Au-Mb sample solutions were centrifuged or subjected to one freeze thaw cycle, gold nanoparticle formation was observed [9].

In this work, a facile one-step synthetic scheme is used to prepare monodispersed gold nanoparticles. It is demonstrated that gold nanoparticles can be rapidly formed when hydroquinone (HQ) is provided as a reductant. Mb is principally responsible for the catalyzed reduction of Au (III) and the stabilization of gold nanoparticles. The size of the gold nanoparticle can be tuned by adjustment of the ratio of Au (III) to Mb in solution. This Mb-mediated system may serve for the synthesis of extremely stable, monodispersed gold nanoparticles with a narrow size distribution. The resulting nanoparticles are homogeneous, spherically shaped and highly stable with no aggregation, even months after the reaction.

## 2. Results and Discussion

**Figure 2:**
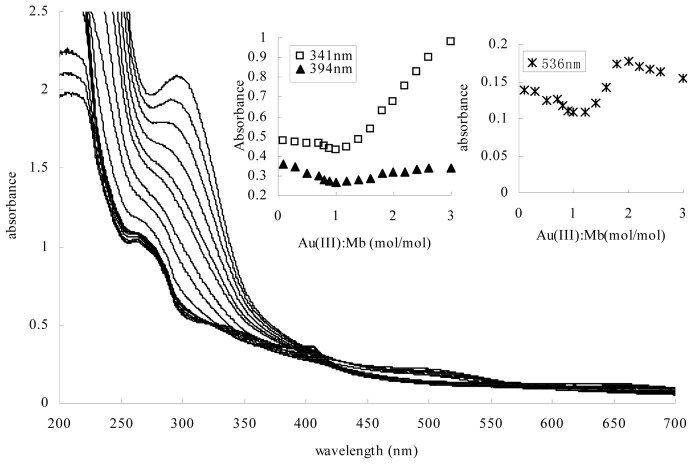
UV-visible absorption spectra of methanobactin (Mb) following the addition of Au (III).

The Mb from *Methylosinus trichosporium* 3011 consists of the Mb from *Methylosinus trichosporium* OB3b in a structure [10,11]. The metal-free form of Mb from *Methylosinus trichosporium* 3011 was light yellow in color, with a weak absorption maxima at 282, 341 and 394 nm (Figure 2). According to the reported UV-visible absorption spectra of Mb from *Methylosinus trichosporium* OB3b, the absorption maximum at 282 nm may be associated with phenolic ion forms of tyrosine. The absorption maxima at 341 nm and 394 nm are associated with the oxazolone ring [12]. Gold coordination experiments were determined by gradual addition of 10 mM solutions of HAuCl_4_ to 0.1 mM aqueous solutions of Mb. At molar ratios of Au (III) to Mb between 0.1 and 1.0, the increases in the absorption maximum at 282 nm and the decreases in the absorption maxima at 341 nm and 394 nm in Mb have been observed with Au (III) addition (Figure 2). It has been reported that the spectral changes were also observed at 282 nm, 341 nm and 394 nm following the addition of Cu (II). The increases in the absorption maxima at 282 nm may represent a charge transfer of phenolic and phenoxide ion forms of tyrosine. The decreases in the absorption maxima at 341 nm and 394 nm suggested the coordination of Cu (II) with the oxazolone ring [12]. Consistent with the spectral changes associated with the addition of Cu (II), the addition of Au (III) resulted in a decreased absorption at 341 nm and 394 nm, suggesting Au (III) was also bound via the oxazolone ring moieties of Mb. The increases in the absorption maxima at 282 nm indicate that the tyrosine of the Mb molecule may contribute to the reducing of Au (III). Further, at molar ratios of Au (III) to Mb above 1.0, the surface plasmon resonance (SPR) of gold nanoparticles was clearly visible as a peak in the range between 530 and 550 nm. Furthermore, an adverse response to Au (III) addition was observed at 341 nm and 394 nm, where an increase in absorbance occurred with Au (III) concentration increase. Examination of the Au (III) and Mb mixture by X-ray photoelectron spectroscopy (XPS) showed the Au (0) 4f7/2 signal at approximately 83.8 eV when Au (III) to Mb ratios were from 0.1 to 3.0. This suggested that at low ratios of Au (III) to Mb, Mb binds and reduces Au (III) to Au (0), but there is little to no formation of gold nanoparticles. At ratios of Au (III) to Mb above 1.0, Mb binds and reduces Au (III) catalytically to Au (0) and yields gold nanoparticles.

**Figure 3:**
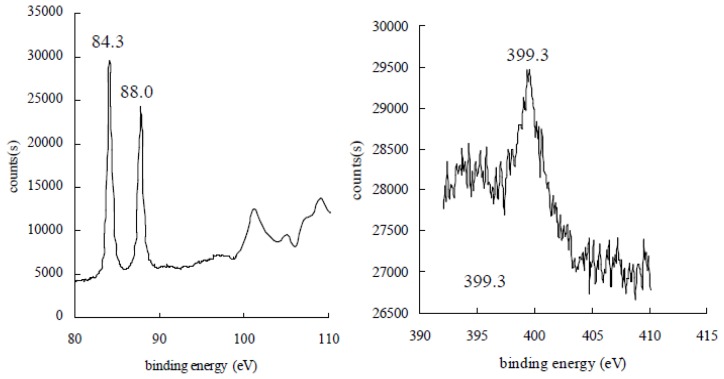
XPS spectra of Au (**left**) and N (**right**) in gold nanoparticles synthesized by Mb.

According to the reports [13], the binding energies of metallic Au (0) 4f7/2 and Au (0) 4f5/2 are 83.8 eV and 87.7 eV, and the binding energies of Au 4f7/2 for the oxidized Au (III) are 86.5 eV. As shown in Figure 3, the X-ray photoelectron spectroscopy (XPS) of the gold nanoparticle samples has two gold signals, one at approximately 84.3 eV, which has been assigned to 4f7/2, and one at 88.0 eV, which has been attributed to 4f5/2. The binding energy of Au 4f7/2 is a little higher than that of the bulk gold at 83.8 eV, indicating the presence of Au (III) on the surface of gold nanoparticles. One nitrogen signal at approximately 399.3 eV was also observed in gold nanoparticle samples. This result suggested that the Au (III) was reduced to Au (0), but not Au (I), by Mb, and Mb-Au (III) chelation may attach on the surface of gold nanoparticles.

**Figure 4:**
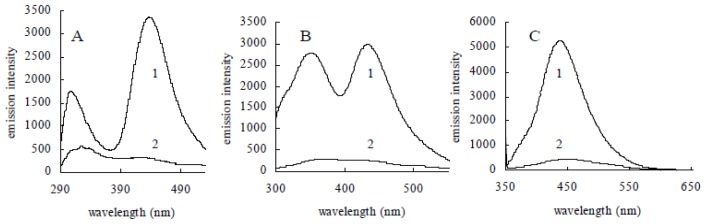
Emission spectra of Mb in aqueous solution with an excitation wavelength of 275 nm (**A**), 280 nm (**B**) and 335 nm (**C**), before (1) and after (2) the addition of HAuCl_4_.

As we all know, gold nanoparticles possess high fluorescent quenching efficiency through both energy-transfer and electron-transfer processes. The fluorescence can be completely quenched via efficient nonradiative fluorescence resonance energy transfer to the gold particle when fluorescein molecules were attached to the surface of gold nanoparticles [14]. Mb molecules were fluorescent in aqueous solution [12]. The fluorescence spectra of Mb from *Methylosinus trichosporium* 3011 showed the characteristic emissions at 314 nm and 432 nm when excited at 275 nm and 280 nm, respectively. A broad emission maximum at 435 nm was also observed following excitation at 335 nm (Figure 4A). Gold coordination experiments were determined by the addition of 10 mM solutions of HAuCl_4_ to 0.1 mM aqueous solutions of Mb. Fluorescence spectra have been monitored followed by the addition of HAuCl_4_ and incubation for 5 min. As shown in Figure 4B, the fluorescence was quenched with the addition of Au (III) to 0.3 mM. The results suggest that Mb molecules were statically adsorbed onto the surface of gold nanoparticles to form a Mb-gold nanoparticle assembly.

**Figure 5:**
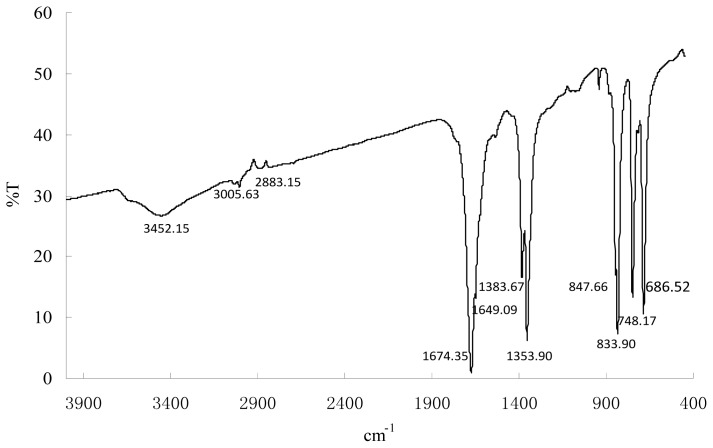
FT-IR spectra of gold nanoparticles synthesized by Mb.

To further demonstrate the presence of capping Mb molecules on the surface of the gold nanoparticles, FT-IR analyses were performed. The experiments revealed the presence of vibration bands centered at 3005.63, 2883.15, 1674.35, 1649.09, 1383.67, 1353.90, 847.66, 833.90, 748.17 and 686.52 along with an intense broad band at 3452.15 cm^−1^ (Figure 5). The broad intense band at about 3452.15 cm^−1^ results from stretch vibrations of H-bonded hydroxyl groups and the N–H stretch of secondary amides. Weaker bands at 3005.63 and 2883.15 cm^−1^ can be attributed to the C–H stretch of aliphatic CH_3_ and CH_2_. The band at 1674.35 cm^−1^ may result from C=O stretching of carboxyl group and ketones, and 1649.09 cm^−1^ may be assigned to C=O and amide (amide I band) stretching. These bands clearly implied the presence of peptide on the nanoparticle surface. The slight shift in the stretching frequency results from significant interaction between the Mb molecule and the gold nanoparticle surface. In fact, all gold nanoparticles need some kind of stabilizing ligand or polymer. These Mb molecules act as surface coating molecules, which prevent the internal agglomeration of the gold nanoparticles. Consequently, the gold nanoparticles were extremely stable in nano-solutions and resisted aggregation, even after several months. (No significant aggregation of the colloid occurred, and the maximum absorption was at ~536 nm without significant red shift.)

**Figure 6:**
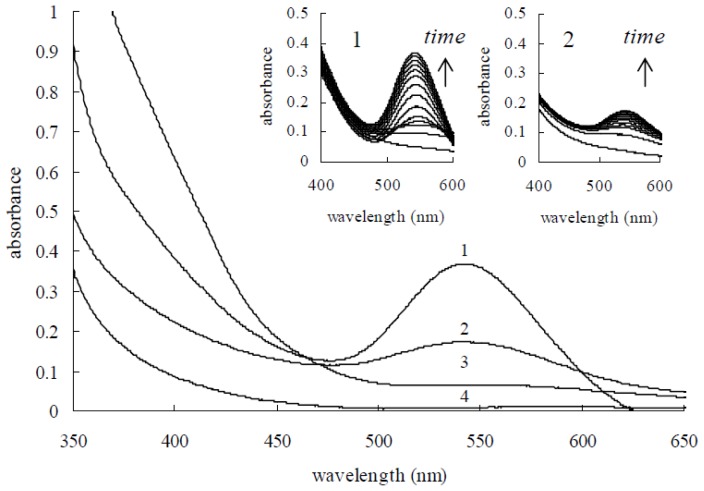
UV-visible spectra recorded for the formation of gold nanoparticles. UV-visible spectra recorded for a solution containing: (**1**) 0.10 mM Mb, 0.50 mM HAuCl_4_ and 0.75 mM Hydroquinone; (**2**) 0.10 mM Mb and 0.50 mM HAuCl_4_; (**3**) 0.50 mM HAuCl_4_ and 0.75 mM Hydroquinone; and (**4**) 0.50 mM HAuCl_4_. Lines 3 and 4 have not any change with time, so no enlarged drawings are inserted.

UV-visible absorption spectroscopy is a convenient way to examine the size and shape of the nanoparticles (NPs) in aqueous suspensions by surface plasmon resonance (SPR) [15]. Herein, we studied the surface plasmon resonance absorption (SPR) spectra using a UV-visible spectrophotometer. The absorption spectra of the mixtures of Mb and HAuCl_4_ solutions were recorded every 10 min for 3 h, and results are illustrated in Figure 6. A steady increase in the absorbance intensity of the surface plasmon resonance feature at 539 nm as a function of the time of the reaction without any major shift in the maximum wavelength can be seen. However, UV-visible spectra of the reaction solution showed that the intensity of the characteristic surface plasmon resonance band for gold nanoparticles centered on 539 nm was weak. The results indicate that Mb has a limited Au (III) reduction capacity and may only reduce limited Au (III) ions to Au (0) in the absence of additional reductant. This limited gold nanoparticle synthesis capacity can be ascribed to the limitation in the amount of reducing power available to reactivate Mb. Continuous synthesis of gold nanoparticles by Mb can be achieved if additional reducing power is provided. It has been reported that Mb showed oxidase activity with hydroquinone (HQ) as the reductant [10]. Mb has also been shown to increase electron flow to the Cu (II) centers of particulate methane monooxygenase (pMMO) and to have superoxide dismutase activity [16]. To retain the gold nanoparticle synthesis capacity of Mb, hydroquinone was used as an external reductant. It is generally acknowledged that hydroquinone is unable to reduce isolated Au (III) ions in acidic and neutral solution [3]. Figure 6 demonstrates that if hydroquinone is the sole agent, it is unable to react with Au (III) on its own; there is no visible surface plasmon resonance peak at 539 nm (curve 3 in Figure 6), even after 24 h. However, if Mb is firstly put into the HAuCl_4_ solution, then hydroquinone is able to reduce Au (III). As shown in Figure 6, the characteristic surface plasmon resonance band for gold nanoparticles centered on 539 nm was increased in intensity with the proceeding course of the reaction. The evolution of the absorbance spectra emanating from gold nanoparticles over time obviously revealed that the production of gold nanoparticles finished within 90 min after exposing the hydroquinone to this HAuCl_4_ and Mb solution.

**Figure 7:**
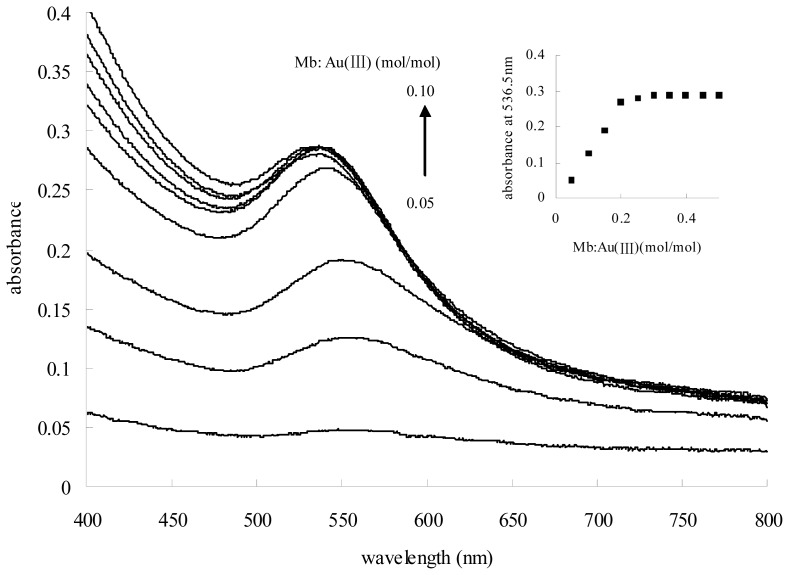
The effect of changing the ratio of Mb to Au (III) on the absorbance of gold nanoparticle solution. Solution containing 0.50 mM HAuCl_4_, 0.75 mM hydroquinone and varying amounts of Mb.

Gold nanoparticles exhibit strong plasmon resonance absorption, which is dependent on the particle size and shape. We synthesized ten gold nanoparticle batches using a consistent concentration of HAuCl_4_ and hydroquinone, but increasing the number of Mb. As expected, the ratio of Mb to Au (III) correlates to the surface plasmon resonance of gold nanoparticle formation. Upon increasing the molar ratio of Mb to Au (III) from 0.05 to 0.25, while keeping the HAuCl_4_ and hydroquinone concentrations constant, an increase in the intensity of the surface plasmon resonance feature with a blue shift from 547.5 nm to 536.0 nm could be observed (Figure 7). However, further increasing of the molar ratio of Mb to Au (III) from 0.30 to 0.50 showed a light enhancement, but no dramatic blue shift, in the surface plasmon resonance feature.

**Figure 8:**
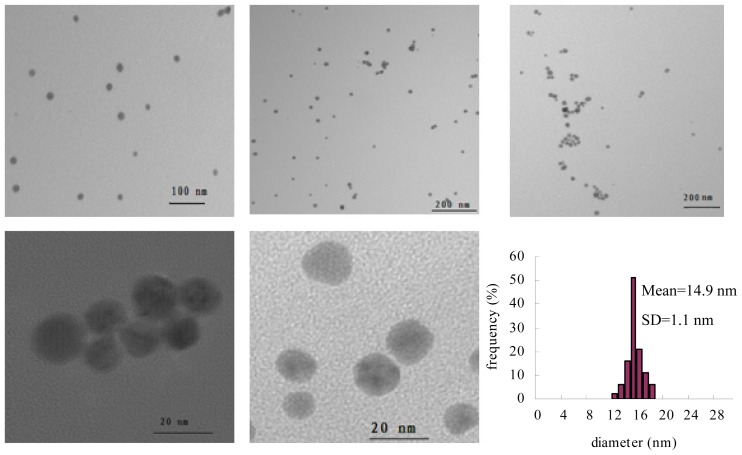
TEM images and size distribution of gold nanoparticles. The first five figures show TEM images of gold nanoparticles at different view areas and their size distribution.

Taking the effect of Mb concentration into consideration, the conditions for gold nanoparticle formation were chosen to be 0.50 mM HAuCl_4_ and 0.75 mM hydroquinone to which is added Mb to a final concentration of 0.15 mM. TEM studies were performed on nanoparticles formed through this protocol. Illustrated in Figure 8 are TEM images of gold nanoparticles formed using the above protocol. The result obtained from the TEM study gave a clear indication regarding the shape and size of the nanoparticles. TEM micrographs of the gold nanoparticles taken at different magnifications showed that the sample is composed of spherical nanoparticles with a mean ratio of 1.13 of longest-to-shortest axes. The gold particles show an average size of 14.9 nm with a standard deviation of 1.1 nm. The relative standard deviation of the gold nanoparticles sizes is smaller than 8%. According to the report by Chen [17] and Kaidanovych [18], the effective monodispersed threshold was defined as a relative standard deviation below 15%. This suggests that the monodispersity of the resulting gold nanoparticles was better.

**Figure 9:**
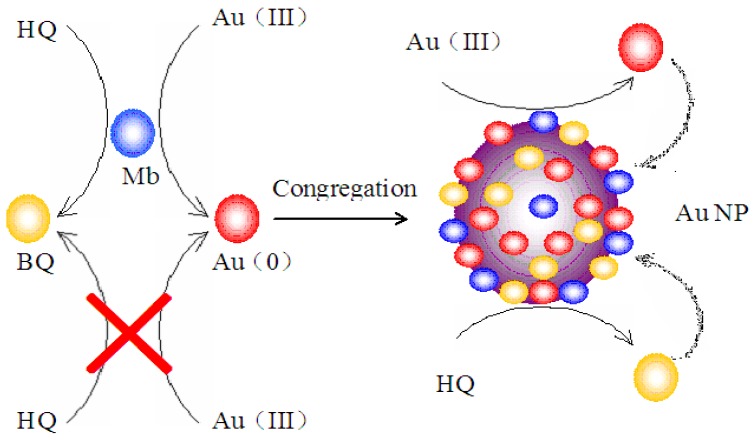
Proposed mechanism of Mb-mediated one-step synthesis of gold nanoparticles. HQ, hydroquinone; BQ, benzoquinone; AuNP, gold nanoparticles.

As shown in Figure 9, the synthesis mechanism may involve Mb-catalyzed reduction of ionic Au (III) to Au (0) and the subsequent Au (III) reduction catalyzed by both gold nanoparticles and the Mb molecules capped on the surface of gold nanoparticles. In the Mb-mediated gold nanoparticle synthesis process, exposure of the Au (III) to Mb creates small Au (0) metallic clusters. Once these nanoparticles or seeds are created, further growth of gold can continue at these particles’ surface through both the Mb-catalyzed and metallic particle surface-catalyzed Au (III) reduction processes with hydroquinone as a reductant. Mb, which forms a capping layer on the gold nanoparticles, is unable to start new particles, even though it continues to grow out of existing particles. Hydroquinone is also unable to reduce isolated Au (III) ions, but is able to reduce those same ions on the surface of metallic clusters. Once the nucleation center is created, hydroquinone can be used on its own to generate additional Au (0) atoms directly on the growing seeds. The monodispersed particle formation can be explained by this forced selectivity, which avoids secondary nucleation.

## 3. Materials and Methods

### 3.1. Organism and Culture Conditions

*Methylosinus trichosporium* 3011 was obtained from the Institute of Microbiology and Virology (Kiev, Ukraine) [19] and was cultivated with a mineral salt medium with the following compositions (g/L) [20]: K_2_HPO_4_ 0.49; KH_2_PO_4_·7H_2_O 0.40; MgSO_4_·7H_2_O 0.30; KNO_3_ 1.6; K_2_SO_4_ 0.17; FeSO_4_·7H_2_O 0.004; CuSO_4_·5H_2_O 0.004; CaCl_2_ ·2H_2_O 0.02; NaCl 0.3; MnSO_4_·5H_2_O 4.0 × 10^−4^; ZnSO_4_·7H_2_O 3.4 × 10^−4^; NaMoO_4_·2H_2_O 2.4 × 10^−4^, pH 7.0. CuSO_4_·5H_2_O was omitted from the medium. Methanol was added to 0.2% (*v*/*v*) and supplied on-line to keep the same concentration. Cells were grown at 28–30 °C and an agitation rate of 250–300 rpm. Ambient air was bubbled through the fermenter continuously at 0.5–0.8 L/min. The cultures were grown to the stationary phase for Mb production.

### 3.2. Isolation of Mb from Spent Media

Mb from the spent medium of *Methylosinus trichosporium* 3011 was isolated as previously described for *Methylococcus capsulatus* Bath by Choi *et al.* [21]. The cells were removed by centrifugation at 10,000× *g* for 30 min. The supernate was loaded onto a 2.5 × 20 cm Diaion HP-20 column (Mitsubishi Chemical Holdings, Tokyo, Japan). The bound Mb was washed with two column volumes of H_2_O and eluted with 30% methanol, 60% H_2_O. The eluant was lyophilized for concentration and storage. The freeze-dried samples following chromatography on Diaion HP-20 columns were the source of Mb used in this study. Typically, 5 to 20 mg of Mb are isolated per liter of spent medium with copper-limited cultures. Freeze-dried samples were stored under moisture-free conditions at −20 °C until enough Mb was collected for the entire experimental program.

### 3.3. Quantification of Mb by Colorimetric Assays

CAS is a chromogenic reagent for Cu (II). The absorbance spectra of the Cu-CAS-HDTMA (hexadecyltrimethylammonium) reagent clearly showed an absorbance peak at 605 nm. When a strong chelator (Mb or EDTA) is added to a highly colored Cu-CAS-HDTMA complex, the release of the copper is accompanied by a 605 nm absorbance peak decrease. Using the ternary complex Cu-CAS-HDTMA as an indicator, both Mb and EDTA exhibit a linear dependence of the absorbance at 605 nm *versus* the concentration of the chelator. The absorbance is directly proportional to the amount of Mb present in the solution, and it can be estimated by comparison with a known chelator standard, such as EDTA [10]. Therefore, the amount of Mb in the sample was estimated using a standard curve of a selected standard EDTA solution.

Chrome azurols (CAS) assay solution was prepared by the following procedure: A 4 mL volume of 0.02 mM HDTMA solution was placed in a 10 mL volumetric flask and diluted with dH_2_O. A mixture of 1 mL Cu solution (0.02 mM CuSO_4_·5H_2_O) and 2 mL 0.02 mM aqueous CAS solution was slowly added under stirring. The volumetric flask was then filled with dH_2_O to afford 10 mL of CAS assay solution. The solutions were stored in the dark.

A 1.0 mL Mb solution was mixed with 9.0 mL-CAS assay solution. A reference control was prepared using 1.0 mL dH_2_O by the same procedure. After reaching equilibrium, the absorbance was measured at 605 nm. All samples were measured in triplicate.

### 3.4. Gold Coordination

Gold coordination was performed using 0.1 mM aqueous solutions of Mb. Stock solution of HAuCl_4_ (10 mM) was added gradually in 0.1 mM aqueous solutions of Mb, mixed and incubated for 5 min before spectral determinations. UV-visible absorption spectroscopy was performed by using a Shimadzu UV-2550 spectrophotometer. Fluorescence excitation spectra were recorded on a Hitachi F-7000 fluorescence spectrometer.

### 3.5. Transmission Electron Microscope (TEM) and X-Ray Photoelectron Spectroscopy (XPS)

The transmission electron microscope (TEM) technique was employed to visualize the size and shape of gold nanoparticles. The samples for TEM were prepared by adding 10 μL of a 1:5 (stock:H_2_O) dilute solution of the sample onto carbon-coated copper grids and allowing water to completely evaporate. TEM images were recorded in a Hitachi H-7650 transmission electron microscope operated at an accelerated voltage of 100 kV. Particle sizes were determined by the ImageJ Java program using the “Particle Analyzer” function. The valence states of elements were analyzed by using X-ray photoelectron spectroscopy (ESCALAB210, VG Scientific, East Grinstead, UK). The samples for XPS were prepared by adding 0.2 mL of aqueous solution of the Au (III) and Mb mixture or 0.2 mL dispersed solution of gold nanoparticles onto a glass plate and allowing the water to completely evaporate. The dispersed solution of gold nanoparticle samples for XPS was prepared by centrifuging the synthesized gold nanoparticles solution at 15,000 rpm for 10 min. The pellet, which contains gold nanoparticles, was redispersed with deionized water three times to get rid of the unattached molecules.

### 3.6. Fourier Transforms Infrared Spectroscopy (FT-IR) Measurements

The samples were analyzed on a PE Spectrum Two FT-IR Spectrometer. A gold nanoparticle powder sample was prepared by centrifuging the synthesized gold nanoparticle solution at 15,000 rpm for 10 min. The pellet, which contains gold nanoparticles, was redispersed with deionized water three times to get rid of the unattached molecules that were not capping ligands for the gold nanoparticles. Then, 0.2–0.5 mg of the dried sample material were ground with 300 mg dried KBr and pressed into a pellet. A background spectrum was recorded with a pellet containing 300 mg KBr.

## 4. Conclusions

The development of techniques for the synthesis of nanoparticles of well-defined size, shape and composition is a challenge and an important area of research in nanotechnology. In this study, we report the biosynthesis of monodispersed gold nanoparticles using Mb as the catalyst and stable agent without the need for any additive for protecting nanoparticles from aggregation. Under the conditions employed in the synthesis, it is clear that hydroquinone is unable to reduce Au (III) ions to gold nanoparticle by itself. However, the formation of gold nanoparticle can be induced by a Mb molecule. The experiments incorporated in this study demonstrate some of the unique properties that can be attributed to using Mb as the catalyst and hydroquinone as a reducing agent when preparing gold nanoparticles. Firstly, Mb shows continuous Au (III) reduction capacity with hydroquinone as the electron donor. Secondly, hydroquinone is able to reduce Au (III) onto metallic particles that are already present, but is unable to reduce Au (III) when the ions are isolated in solution. Thirdly, the gold nanoparticles are stable in solution, because the present Mb capping layer prevents particle aggregation. Gold nanoparticle formation can seemingly be stopped at a desired nanoparticles size by adjusting the ratio of Au (III) to Mb. In general, at Au (III) to Mb ratios of about three, the average size of the gold nanoparticles is about 14.9 nm with a standard deviation of 1.1 nm. The UV-visible absorption spectra of gold nanoparticles exhibited a symmetric spectrum with a surface plasmon resonance (SPR) peak at 536 nm. These TEM data and UV-visible spectral analysis confirm that the gold nanoparticles are monodispersed upon synthesis. To further verify the monodispersity, the estimated size of gold nanoparticles based on the powder X-ray diffraction (XRD) analysis will be done in future work. In conclusion, this simple technique produces monodispersed gold nanoparticles in a reproducible way. Future work will focus on the effect of synthesis temperatures on the morphology.

## References

[1] Daniel M.C., Astruc D. (2004). Gold nanoparticles: Assembly, supramolecular chemistry, quantum-size-related properties, and applications toward biology, catalysis, and nanotechnology. Chem. Rev.

[2] Sardar R., Funston A.M., Mulvaney P., Murray R.W. (2009). Gold nanoparticles: Past, present and future. Langmuir.

[3] Perrault S.D., Chan W.C.W. (2009). Synthesis and surface modification of highly monodispersed, spherical gold nanoparticles of 50–200 nm. J. Am. Chem. Soc.

[4] Rao C.N.R., Cheetham A.K. (2001). Science and technology of nanomaterials: Current status and future prospects. J. Mater. Chem.

[5] Sau T.K., Pal A., Jana N.R., Wang Z.L., Pal T. (2001). Size controlled synthesis of gold nanoparticles using photochemically prepared seed particles. J. Nanopart. Res.

[6] Mallick K., Wang Z.L., Pal T. (2001). Seed-mediated successive growth of gold particles accomplished by UV irradiation: A photochemical approach for size-controlled synthesis. J. Photoch. Photobio. A.

[7] Hanson R.S., Hanson T.E. (1996). Methanotrophic bacteria. Microbiol. Rev.

[8] Balasubramanian R., Rosenzweig A.C. (2008). Copper methanobactin: A molecule whose time has come. Curr. Opin. Chem. Biol.

[9] Choi D.W., Do Y.S., Zea C.J., McEllistrem M.T., Lee S.W., Semrau J.D., Pohl N.L., Kisting C.J., Scardino L.L., Hartsel S.C. (2006). Spectral and thermodynamic properties of Ag(I), Au(III), Cd(II), Co(II), Fe(III), Hg(II), Mn(II), Ni(II), Pb(II), U(IV), and Zn(II) binding by methanobactin from *Methylosinus trichosporium* OB3b. J. Inorg. Biochem.

[10] Xin J.Y., Jiang J.L., Zhang S., Guan H.N., Chen L.L., Xia C.G. (2013). Oxidation of hydroquinone catalyzed by methanobactin-Cu with hydrogen peroxide. Chem. J. Chin. Univ.

[11] Hakemian A.S., Tinberg C.E., Kondapalli K.C., Telser J., Hoffman B.M., Stemmler T.L., Rosenzweig A.C. (2005). The copper chelator methanobactin from Methylosinus trichosporium OB3b binds copper(I). J. Am. Chem. Soc.

[12] Choi D.W., Zea C.J., Do Y.S., Semrau J.D., Antholine W.E., Hargrove M.S., Pohl N.L., Boyd E.S., Geesey G.G., Hartsel S.C. (2006). Spectral, kinetic, and thermodynamic properties of Cu(I) and Cu(II) binding by methanobactin from methylosinus trichosporium OB3b. Biochemistry.

[13] Huang J., Dai W.L., Fan K.N. (2009). Remarkable support crystal phase effect in Au/FeO*_x_* catalyzed oxidation of 1,4-butanediol to γ-butyrolactone. J. Catal.

[14] Guo L., Zhong J., Wu J., Fu F., Chen G., Chen Y., Zheng X., Lin S. (2011). Sensitive turn-on fluorescent detection of melamine based on fluorescence resonance energy transfer. Analyst.

[15] Wiley B.J., Im S.H., McLellan J., Seikkinen A., Xia Y. (2006). Maneuvering the surface plasmon resonance of silver nanostructures through shape-controlled synthesis. J. Phys. Chem. B.

[16] Choi D.W., Semrau J.D., Antholine W.E., Hartsel S.C., Anderson R.C., Carey J.N., Dreis A.M., Kenseth E.M., Renstrom J.M., Scardino L.L. (2008). Oxidase, superoxide dismutase, and hydrogen peroxide reductase activities of methanobactin from types I and II methanotrophs. J. Inorg. Biochem.

[17] Chen S.L., Yuan G.M., Hu C.T. (2011). Preparation and size determination of monodisperse silica microspheres for particle size certified reference materials. Powder Technol.

[18] Kaidanovych Z., Kalishyn Y., Strizhak P. (2013). Deposition of monodisperse platinum nanoparticles of controlled size on different supports. Adv. Nanopart.

[19] Xin J.Y., Cui J.R., Chen J.B., Li S.B., Xia C.G., Zhu L.M. (2003). Continuous biocatalytic synthesis of epoxypropane using a biofilm reactor. Proc. Biochem.

[20] Xin J.Y., Zhang Y.X., Dong J., Zhou Q.Q., Wang Y., Zhang X.D., Xia C.G. (2010). Epoxypropane biosynthesis by whole cell suspension of methanol-growth *Methylosinus trichosporium* IMV 3011. World J. Microbiol. Biotechnol.

[21] Choi D.W., Antholine W.E., Do Y.S., Semrau J.D., Kisting C.J., Kunz R.C., Campbell D., Rao V., Hartsel S.C., DiSpirito A.A. (2005). Effect of methanobactin on the activity and electron paramagnetic resonance spectra of the membrane-associated methane monooxygenase in methylococcus capsulatus bath. Microbiology.

